# Use of Repeated Group Measurements with Drop Out Animals for Variance Component Estimation and Genetic Evaluation: A Simulation Study

**DOI:** 10.1534/g3.119.400484

**Published:** 2019-07-05

**Authors:** Hongding Gao, Bjarne Nielsen, Guosheng Su, Per Madsen, Just Jensen, Ole F. Christensen, Tage Ostersen, Mahmoud Shirali

**Affiliations:** * Center for Quantitative Genetics and Genomics, Department of Molecular Biology and Genetics, Aarhus University, Tjele 8830, Denmark and; † SEGES, Pig Research Centre, Copenhagen 1609, Denmark

**Keywords:** random regression, longitudinal, group composition, feed intake, accuracy

## Abstract

The efficiency of feed utilization plays an important role in animal breeding. However, measuring feed intake (FI) is costly on an individual basis under practical conditions. Using group measurements to model FI could be practically feasible and cost-effective. The objectives of this study were to develop a random regression model based on repeated group measurements with consideration of missing phenotypes caused by drop out animals. Focus is on variance components (VC) estimation and genetic evaluation, and to investigate the effect of group composition on VC estimation and genetic evaluation using simulated datasets. Data were simulated based on individual FI in a pig population. Each individual had measurement on FI at 6 different time points, reflecting 6 different weeks during the test period. The simulated phenotypes consisted of additive genetic, permanent environment, and random residual effects. Additive genetic and permanent environmental effects were both simulated and modeled by first order Legendre polynomials. Three grouping scenarios based on genetic relationships among the group members were investigated: (1) medium within and across pen genetic relationship; (2) high within group relationship; (3) low within group relationship. To investigate the effect of the drop out animals during test period, a proportion (15%) of animals with individual phenotypes was set as the drop out animals, and two drop out scenarios within each grouping scenario were assessed: (1) animals were randomly dropped out; (2) animals with lower phenotypes were dropped out based on the ranking at each time point. The results show that using group measurements yielded similar VCs estimates but with larger SDs compared with the corresponding scenario of using individual measurements. Compared to scenarios without drop out, similar VC estimates were observed when animals were dropped out randomly, whereas reduced VC estimates were observed when animals were dropped out by the ranking of phenotypes. Different grouping scenarios produced similar VC estimates. Compared to scenarios without drop out, there were no loss of accuracies of genetic evaluation for drop out scenarios. However, dropping out animals by the ranking of phenotypes produced larger bias of estimated breeding values compared to the scenario without dropped out animals and scenario of dropping out animals by random. In conclusion, with an optimized group structure, the developed model can properly handle group measurements with drop out animals, and can achieve comparable accuracy of genetic evaluation for traits measured at the group level.

Feed intake (FI) and feed efficiency are commonly measured in repeated periods during productive periods of animals and have been shown to have varying genetic background across the test trajectory in a number of species such as mink (Shirali *et al.* 2015), pigs (Shirali *et al.* 2017), and dairy cattle (Li *et al.* 2017). Under commercial animal breeding practices, for some economically important traits, it may be more cost-effective to measure performance records on group level compared to individual level. Several studies have reflected on the fact that a large number of records are available on group level in many animal breeding programs, for instance FI in cages of mink (Shirali *et al.* 2015), egg production and body weight in groups of laying hens (Peeters *et al.* 2013; Biscarini *et al.* 2010; Biscarini *et al.* 2008).

The use of such records for genetic evaluation were first suggested in a simulation study by Olson *et al.* (2006), who pooled individual records together to represent the joint performance of a group of animals, and demonstrated the efficacy of using group records with the usual mixed model for genetic evaluation. They concluded that group records can be effectively applied in animal genetic evaluation and selection decision despite a loss of evaluation accuracy. Based on real data for body weight from laying hens, Biscarini *et al.* (2008) developed a procedure for estimating genetic parameters and predicting breeding values using group records. Their results demonstrated that, using group records for genetic analyses were both theoretically and practically feasible, provided groups had the same size. Recently, Su *et al.* (2018) further developed the approach of Olson *et al.* (2006) in a simulation study. They concluded that it was possible to extend the approach to account for differing group size, and non-genetic random effects such as litter and pen effect for genetic parameter estimation and breeding value prediction. These approaches for genetic analyses of group records have revealed that group composition and group size are the vital factors influencing the accuracy of genetic parameter estimation and genetic evaluation when using group measurements (Peeters *et al.* 2013; Su *et al.* 2018). However, all these applications of using group measurements were with a single measurement from each group.

In order to effectively use all information in practical breeding programs, it would be attractive to further extend to models for analyzing longitudinal records. A particular concern with group records on longitudinal traits is that animals may drop out from the groups during the test, due to *e.g.*, sickness or death. Hence, such group records at different time points may not represent the same animals.

The objectives of this study were (1) to develop a random regression model based on repeated group measurements with a consideration of drop out animals for estimation of variance components (VCs) and genetic evaluation; and (2) to examine the effect of group composition on estimates of VC and accuracy of predicting breeding values.

## Materials and Methods

### Simulated Data

A dataset based on individual measurements for FI in a pig population was simulated. The simulated population consisted of 11 generations without overlap. In each generation, 30 sires were selected and each sire was mated to 20 dams with a litter size of 6 individuals. For simplicity, random selection and random mating were performed in the simulated population and sows were assumed to have one parity only. Thus, the possible genetic trend across generations was not accounted for in this study. Each individual had 6 measurements on FI at 6 different time points reflecting 6 different weeks during the test period. In this study, the group measurement was defined as the sum of the individual measurements within a group. Consequently, each group had 6 measurements reflecting 6 different time points. The phenotypes from the last 5 generations were used for VCs estimation and genetic evaluation.

**Figure 1:**
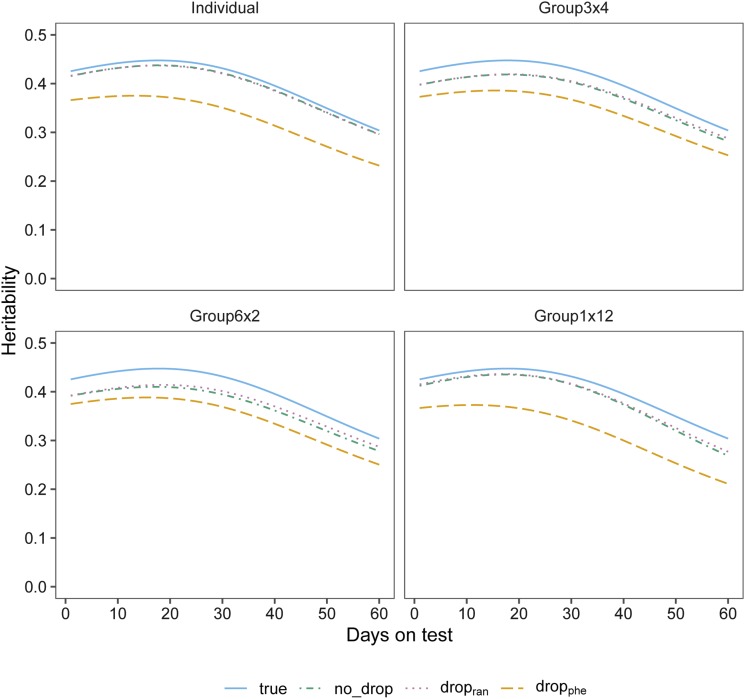
The trajectory of simulated (true) heritability, and heritability estimates (mean of 10 replicates) as a function of days on test for different drop out scenarios within individual measurement and each grouping strategy. No_Drop: measurements without drop out animals; Drop_ran_: drop out animals were randomly selected and the drop out time of each drop out animal was sampled from a vector of the integers from 1 to 6 representing the six testing time points; Drop_phe_: six time intervals were defined based on six testing time points, the number of drop out animals at each interval was sampled from a Poisson distribution with a mean equal to the defined proportion of drop out animals divided by six. Animals were ranked based on their phenotypes at each testing time point, then dropped out based on the number sampled from the Poisson distribution; medium within and across pen genetic relationship (Group_3×4_): group was consisted of 4 different families with 3 pigs from each family; high within group relationship (Group_6×2_): all animals from 2 different litters were allocated to a group; low within group relationship (Group_1×12_): all animals were from different litters.

The simulated phenotypic records consisted of additive genetic, permanent environmental, and residual effects. First order Legendre polynomials were used to generate both additive genetic and permanent environmental effects; hence, additive genetic effects for each animal contained two coefficients, intercept (b_0_) and slope (b_1_). Under such a design, additive genetic effects for the founder animals were drawn directly from a multivariate normal distribution N(0, G), where $G=\left[\begin{array}{cc} 63.42 & -5.42\\ -5.42 & 6.85 \end{array}\right]$ is a 2×2 genetic covariance matrix of the intercept and regression coefficient; thus, the breeding values for founder animals were calculated as $L\left[\begin{array}{c} {\text{b}}_{0}\\ {\text{b}}_{1} \end{array}\right]$, where **L** is a 6×2 matrix of Legendre polynomials for the 6 test time points; additive genetic effects for each offspring were calculated as $a_{i}=\frac{1}{2}\left(a_{s}+a_{d}\right)+m_{i},$ where $a_{i},$ $a_{s}$ and $a_{d}$ are the vector of breeding values for animal *i*, with sire *s* and dam *d*, respectively. The vector $m_{i}$ is the Mendelian sampling terms for animal *i* drawn from a multivariate normal distribution $N\left(0,\;\frac{1}{2}\left(1-F_{i}\right)G\right),$ and $F_{i}=\frac{1}{2}(F_{s}\;+\;F_{d})$; where *F_s_* and *F_d_* are the inbreeding coefficients of sire *s* and dam *d* for animal *i*, respectively. Permanent environmental effects were drawn from a multivariate normal distribution with mean of zero and variance of $P=\;\left[\begin{array}{cc} 30.61 & 4.28\\ 4.28 & 29.25 \end{array}\right]$. The heritabilities ($h^{2}$) assumed along the testing time trajectory are shown in Figure 1. By assuming a homogeneous residual variance in the simulation, residual effect for each animal was drawn from a normal distribution with mean of zero and variance of $\sigma_{e}^{2}=53.45$. It was assumed that there were no interactions among these effects.

Individual phenotype for each animal at each time point on the test was obtained as $y_{t}=\emptyset_{t}a_{t}+\emptyset_{t}p_{t}+{\text{e}}_{t}$ where $\emptyset_{t}$ is a 1×2 matrix coefficients of Legendre polynomials at time point *t*; **a**_t_ and **p**_t_ are 2×1 matrices of sampled intercept and regression coefficient of each animal for additive genetic and permanent environmental effects, respectively; e_t_ is a scalar of sampled residual. A pen was constructed by allocating 12 animals to a group unit.

### Group Composition Scenarios

Three grouping scenarios based on genetic relatedness of the group members were investigated for VCs estimation and genetic evaluation: (1) medium within and across pen genetic relationship (Group_3×4_): a litter was randomly split into two sub-litters of size 3, group was consisted of 4 different sub-litters (families); (2) high within group relationship (Group_6×2_): all pigs from 2 different litters were allocated to a group; (3) low within group relationship (Group_1×12_): group consisted of 12 pigs, which were randomly selected from 12 different litters. The grouping process was conducted within generation.

To investigate the effect of the drop out animals during test period, a proportion of animals (15%) from the last five generations (since only phenotypes of animals from the last five generations were used for analysis) was set as the drop out animals. Once the animal was dropped out, the phenotypes of that animal from the drop out time point and forward were set to missing. In the present study, two drop out strategies within each grouping scenario were assessed: (1) Drop_ran_: drop out animals were randomly selected, and the drop out time of each drop out animal was sampled randomly from a vector of the integers from 1 to 6 representing the six testing time points; (2) Drop_phe_: six time intervals were defined based on six testing time points, the number of drop out animals at each interval was sampled from a Poisson distribution with a mean equal to the total size of drop out animals divided by six. Animals were ranked from low to high level based on their phenotypes at each testing time point, then a number of animals with lowest of phenotypes were dropped out. The number of drop out animals was based on the number sampled from the Poisson distribution. For each scenario, we performed 10 replicates.

The simulation was conducted in R (R Core Team 2019) and Julia (Bezanson *et al.* 2017). The scripts are in supplemental files.

### Statistical models

Repeated individual measurements were analyzed by random regression model as follows:

$$y_{it}=\sum_{k=0}^{nf}\emptyset_{itk}\beta_{k}+\sum_{k=0}^{nr}\emptyset_{itk}a_{ik}+\sum_{k=0}^{np}\emptyset_{itk}pe_{ik}+e_{it}$$

where $y_{it}$ were the phenotype of animal *i* measured on time point *t*; $\beta_{k}$ is the *k*^th^ fixed regression coefficient; $a_{ik}$ and $pe_{ik}$ are the *k*^th^ random regression coefficients of animal *i* for additive genetic and permanent environmental effects, respectively; $\emptyset_{itk}$ is the Legendre polynomials as the covariates for the record of animal *i* measured on time point *t*; *nf* is the order of polynomials fitted as fixed regressions; *nr* is the order of polynomials for additive genetic effects; *np* is the order of polynomials for permanent environment effect, *nf*, *nr*, and *np* were all first order Legendre polynomials; $e_{it}$ is the random residual with assumption of homogeneous variance. It was assumed that the additive genetic effects follow N(0, A⊗G), the permanent environmental effects follow N(0, I⊗P), and the residual effects follow $N\left(0,\;I\sigma_{e}^{2}\right),$ where **A** is the numerator relationship matrix, and **I** is the identity matrix.

Repeated group measurements were analyzed by the following random regression model obtained by summing the terms in the model for individual measurements within each group *j*, and is as follows:

$$y_{jtm_{j}}^{*}=GS_{m_{j}}\sum_{k=0}^{nf}\emptyset_{jtk}\beta_{jk}+\sum_{i=1}^{n_{jt}}\sum_{k=0}^{nr}\emptyset_{ijtk}a_{ijk}+\sum_{i=1}^{n_{jt}}\sum_{k=0}^{np}\emptyset_{ijtk}pe_{ijk}+e_{jtm_{j}}^{*}$$

where $y_{jtm_{j}}^{*}$ is the phenotype of group *j* measured at time *t* with group size (*GS*) of *m* (*m* = 1, 2, …... 12); $GS_{m_{j}}$ is the fixed effect of group size *m* for group *j*; $\beta_{jk}$ is the *k*^th^ fixed regression coefficient for group *j* nested within group size effect *m*; $a_{ijk}$ and $pe_{ijk}$ are the *k*^th^ random regression for additive genetic and permanent environmental effects, respectively, for animal *i* in group *j*; $\emptyset_{ijtk}$ is the covariate coefficient of Legendre polynomials for animal *i* in group *j* measured at time *t*; $n_{jt}$ is the number of animals in group *j* at time *t*; $e_{jtm_{j}}^{*}$ is the random residual with the variance of $D\sigma_{e}^{2}$, where **D** is a diagonal matrix with elements of group sizes for group *j*.

Variance components were estimated by restricted maximum likelihood (REML) (Patterson and Thompson 1971) with the average information algorithm (AI-REML) (Gilmour *et al.* 1995; Johnson and Thompson 1995; Madsen *et al.* 1994), breeding values were predicted by best linear unbiased prediction (BLUP) (Henderson 1985). All analyses were performed using the DMU package (Madsen and Jensen 2013).

### Validation

A selection index of FI was defined in this study. Based on the characteristics of longitudinal records, where performance is measured sequentially over time on each animal. The FI measurements for all 6 time points during the test phase can be considered as 6 different and correlated traits. The selection index was then calculated as a weighted sum of the estimated breeding values (EBV) from all the 6 testing time points using weight of 1 for each trait. To compare the efficiency of using group measurements with individual measurements, accuracy of genetic evaluation was computed as the Pearson correlation coefficient between EBVs and simulated breeding values for animals having phenotypic data. Unbiasedness of genetic evaluation was evaluated through the regression of simulated (true) breeding values on the EBVs for animals having phenotypic data.

### Data Availability

Simulated data available at Figshare: https://doi.org/10.6084/m9.figshare.8047367.

Files at this URL are simulated pedigree and phenotypes based on different grouping scenarios and drop out strategies. Simulation scripts available at Figshare: https://doi.org/10.6084/m9.figshare.8312555.

The authors affirm that all data necessary for confirming the conclusions of the article are present within the article, and tables. Supplemental material available at FigShare: https://doi.org/10.25387/g3.8046995.

## Results

Table 1 presents the mean and standard deviation (SD) of estimated VCs over 10 replicates using repeated individual and group measurements based on different grouping and drop out scenarios. Unbiased estimates of VCs were obtained when analyzing individual measurements. In general, using group measurements yielded similar VCs estimates but with larger SDs as the corresponding scenario with individual measurements. Similar VC estimates were observed from random drop out scenarios (Drop_ran_) compared with no drop out scenario (No_Drop). Different grouping scenarios based on genetic relatedness of the group members produced similar VCs estimation. The grouping scenario of high within group relationship (Group_6×2_) resulted in lowest SDs for genetic (co)variances and residual variance among all the grouping scenarios. However, the SDs for permanent environmental (co)variances were the highest among all the grouping scenarios.

**Table 1:** Mean and standard deviation (SD) of estimated variance components (VC) of genetic (G_ij_), permanent environmental (P_ij_) and residual effects ($\sigma_{e}^{2}$) over 10 replicates from repeated individual measurements and group measurements based on different grouping and drop out scenarios^1^

		Individual			Group_3×4_			Group_6×2_			Group_1×12_		
VC^2^	Simulated	No_Drop	Drop_ran_	Drop_phe_	No_Drop	Drop_ran_	Drop_phe_	No_Drop	Drop_ran_	Drop_phe_	No_Drop	Drop_ran_	Drop_phe_
**G**_11_	63.42	61.37 (2.15)	61.18 (2.36)	42.40 (1.95)	58.72 (7.79)	58.93 (7.15)	45.66 (7.88)	57.62 (3.62)	58.24 (4.41)	45.80 (3.34)	60.15 (8.21)	60.68 (8.85)	41.85 (7.15)
**G**_12_	−5.42	−5.22 (0.78)	−5.24 (0.78)	−5.44 (0.84)	−5.23 (1.55)	−5.04 (1.71)	−5.14 (1.76)	−5.11 (1.60)	−4.80 (1.70)	−5.24 (1.24)	−6.33 (3.05)	−5.95 (2.95)	−6.19 (3.10)
**G**_22_	6.85	6.63 (0.80)	6.74 (0.78)	5.70 (0.74)	6.21 (1.78)	6.61 (1.96)	5.56 (2.04)	6.30 (1.00)	6.31 (0.92)	5.29 (1.08)	5.14 (1.78)	5.80 (1.74)	4.92 (1.57)
**P**_11_	30.61	31.15 (1.07)	31.28 (1.10)	28.15 (0.95)	33.71 (5.62)	33.56 (5.60)	28.16 (5.59)	35.73 (7.06)	34.01 (6.47)	28.28 (3.90)	30.92 (4.71)	31.38 (5.53)	30.13 (4.80)
**P**_12_	4.28	4.26 (0.48)	4.34 (0.59)	2.77 (0.43)	3.80 (2.80)	3.52 (1.72)	2.14 (2.87)	3.84 (3.45)	3.34 (3.23)	2.41 (2.03)	5.00 (2.71)	5.13 (2.00)	3.38 (2.97)
**P**_22_	29.25	29.36 (0.64)	29.21 (0.63)	25.92 (0.64)	29.60 (2.38)	29.45 (2.75)	25.45 (2.04)	29.27 (2.94)	29.27 (3.49)	25.38 (2.65)	30.32 (2.61)	29.95 (2.41)	25.22 (1.85)
$\sigma_{e}^{2}$	53.45	53.47 (0.33)	53.47 (0.33)	50.92 (0.34)	53.37 (1.03)	53.30 (1.03)	50.64 (0.99)	53.42 (0.64)	53.46 (0.67)	50.53 (0.48)	53.93 (0.77)	53.81 (0.85)	50.98 (0.88)

1 Medium within and across pen genetic relationship (Group_3×4_): group was consisted of 4 different families with 3 pigs from each family; High within group relationship (Group_6×2_): all animals from 2 different litters were allocated to a group; Low within group relationship (Group_1×12_): all animals were from different litters. No_Drop: measurements without drop out animals; Drop_ran_: drop out animals were randomly selected and the drop out time of each drop out animal was sampled from a vector of the integers from 1 to 6 representing the six testing time points; Drop_phe_: six time intervals were defined based on six testing time points, the number of drop out animals at each interval was sampled from a Poisson distribution with a mean equal to the total size of drop out animals divided by six. Animals were ranked based on their phenotypes at each testing time point, then dropped out based on the number sampled from the Poisson distribution.

2 **G**(**P**)_11_: variance of intercept; **G**(**P**)_12_: covariance between intercept and slope; **G**(P)_22_: variance of slope.

Figure 1 presents a multi-panel plot showing the trajectory of simulated (true) heritability, and heritability estimates (mean of 10 replicates) as a function of days on test for different drop out scenarios within each grouping strategy. Clearly, the heritabilities were not constant throughout the test period with a tendency toward lower heritability estimates in the middle and late stages of test period. This trend was consistent across different grouping strategies. The trajectories of heritability estimates were similar between no drop out scenario (No_Drop) and random drop out scenarios (Drop_ran_). Lowest estimates of heritability were observed for scenario of Drop_phe_.

Mean and SD of evaluation accuracy and bias using repeated individual and group measurements based on different grouping and drop out scenarios are shown in Table 2. As expected, the highest accuracy was achieved by using individual measurements. As can be seen from the table, compared with no drop out scenarios, drop out scenarios show that there is no loss of evaluation accuracy. Among the grouping scenarios, the high within group relationship (Group_6×2_) had highest accuracy, whereas the low within group relationship (Group_1×12_) had lowest accuracy. Within drop out scenarios, random drop out scenarios (Drop_ran_) and scenarios of drop out by phenotypes (Drop_phe_) resulted in similar accuracies. Unbiased evaluations reflected by regression of simulated on predicted genetic values, were obtained when using individual measurements and group measurements from no drop out grouping scenarios (No_Drop) and random drop out scenarios (Drop_ran_). Deflation of EBVs from scenarios of drop out by phenotypes (Drop_phe_) were found across all the scenarios.

**Table 2:** Mean and standard deviation (SD) of prediction and bias of estimated breeding values (EBV) over 10 replicates from repeated individual measurements and group measurements based on different grouping and drop out scenarios^1^

	Individual			Group_3×4_			Group_6×2_			Group_1×12_		
Item^2^	No_Drop	Drop_ran_	Drop_phe_	No_Drop	Drop_ran_	Drop_phe_	No_Drop	Drop_ran_	Drop_phe_	No_Drop	Drop_ran_	Drop_phe_
r	0.858 (0.019)	0.859 (0.019)	0.829 (0.023)	0.676 (0.052)	0.677 (0.050)	0.671 (0.052)	0.707 (0.043)	0.707 (0.043)	0.701 (0.044)	0.629 (0.058)	0.635 (0.060)	0.622 (0.058)
b_1_	1.004 (0.013)	1.006 (0.014)	1.113 (0.023)	1.009 (0.031)	1.005 (0.035)	1.152 (0.052)	1.019 (0.031)	1.016 (0.032)	1.149 (0.038)	0.990 (0.019)	0.992 (0.020)	1.180 (0.055)

1 Medium within and across pen genetic relationship (Group_3×4_): group was consisted of 4 different families with 3 pigs from each family; High within group relationship (Group_6×2_): all animals from 2 different litters were allocated to a group; Low within group relationship (Group_1×12_): all animals were from different litters. No_Drop: measurements without drop out animals; Drop_ran_: drop out animals were randomly selected and the drop out time of each drop out animal was sampled from a vector of the integers from 1 to 6 representing the six testing time points; Drop_phe_: six time intervals were defined based on six testing time points, the number of drop out animals at each interval was sampled from a Poisson distribution with a mean equal to the total size of drop out animals divided by six. Animals were ranked based on their phenotypes at each testing time point, then dropped out based on the number sampled from the Poisson distribution.

2 r: Pearson correlation coefficient between EBVs and simulated breeding values for animals having phenotypic data; b_1_: regression coefficient of simulated breeding values on EBVs for animals having phenotypic data.

## Discussion

In the present study, a random regression model for analyzing repeated group measurements was developed and investigated on simulated data. The model was able to consider varying group sizes during the test period caused by drop out animals, which addresses an important practical issue. Different grouping strategies in terms of genetic relatedness among the group members were also assessed. The results show that the random regression model for longitudinal group measurements yielded unbiased VCs estimation for scenarios of random drop out, and the high within group relationship (Group_6×2_) achieved the highest accuracy among all the grouping scenarios.

### Group *vs.* individual measurements

The SDs of estimated VCs from group measurements were larger compared with those from individual measurements. This indicates less information was available due to merging individual measurements into group measurements. Larger SE of genetic parameter estimates was also reported by (Biscarini *et al.* 2008) when group analysis of body weight on cage level was compared to individual body weight recording for laying hens. Peeters *et al.* (2013) used simulated pooled records to estimate VCs for traits affected by social interactions in laying hens and found the VC estimates from group records did not differ significantly from the simulated VCs. The results in the current study is in line with a recent study of Su *et al.* (2018), where VCs estimated from univariate group records were reported to be consistent with those estimated from univariate individual records but with larger standard errors.

The accuracy of EBV is of great importance for animal improvement programs as animals are ranked based on their breeding values. In the current study, we observed lower accuracies when using group measurements compared with using individual measurements. The reduction of evaluation accuracy is as expected and due to the reduction of information content by merging the animals into groups. Olson *et al.* (2006) used simulated group measurements for genetic evaluation, and reported that accuracies of using group measurements compared to those of using individual measurements decreased from 0.77 to 0.50, from 0.77 to 0.53 for random grouped animals and animals grouped based on sires, respectively; the reduction of accuracy were more profound as the group size increased.

Biscarini *et al.* (2008) reported that EBV accuracy obtained from pooled records was about 70–80% of the accuracy of EBV predicted from individual records. Su *et al.* (2018) reported 68% for the EBV accuracy achieved from group records to that from individual records. However, the notable cost savings of using group measurements compared with using individual measurements can counteract the reduced prediction accuracy.

### Group Composition

In the present study, we simulated the trait of FI in a pig population, hence, a pen was defined as a group. In practice, the strategies of allocating pigs into pens varies across herds; therefore, a good grouping strategy can maximize the effective use of group information. In this study, this was demonstrated by the group scenario of Group_6×2_ in terms of highest accuracies of genetic evaluation among all the grouping scenarios, where the animals in the group had the closest genetic relationships compared with scenarios of Group_3×4_ and Group_1×12_. Our results are in line with the previous simulation study by (Olson *et al.* 2006) who found that allocating animals to groups on the basis of their sire information achieved better accuracies of breeding values than allocating animals to groups on the basis of maternal grandsire (MGS) and random allocation. Through theoretical derivations and simulations, Peeters *et al.* (2013) showed that smaller SE on genetic variance estimates were achieved when allocating animals from the same family to the group. Interestingly, when directly using the VCs from simulation, Su *et al.* (2018) verified the accuracy of breeding values were increased with increased genetic relationships of the group members. A possible explanation for these results might be that, when we use the group measurements, the proportion of additive genetic to phenotypic variance increases as the genetic relationships among the group members increase (Su *et al.* 2018). However, it is important to note that one major difference between their studies and the current study is that we used repeated group measurements whereas they used single group measurement.

### Drop outs

The method presented in this study allows consideration of missing phenotypes caused by drop out animals in each group. In particular, the scenario of dropping out animals by ranking of phenotypes (Drop_phe_) was designed to reflect the practical situation, where animals with poor phenotypes are more susceptible to diseases and thus, more likely to be culled. We observed reduced VC estimates from the scenario of Drop_phe_ for both individual and group measurement (Table 1), leading to larger biases of EBV (Table 2), these results were mainly influenced by the decreased phenotypic and genetic variances due to reduced number of animals with lower performance records. Therefore, this seems that, it would be preferable to perform genetic evaluation using group measurements with VCs estimated from individual measurements, if feasible.

### Extra random effects

In the current study, litter and pen effects were not simulated in order to provide a simple platform for explanation of the approach. However, those effects were investigated by

Su *et al.* (2018) based on a univariate analysis (single measurement), where group records were modeled with considering of litter and pen effects, and compared with the models ignoring litter and pen effects. Their results showed that ignoring litter and pen effects had no influence on the prediction accuracy. Extending the use of the current model to include extra random effects can refer to the same methodology as presented by Su *et al.* (2018).

The design of the current simulation study was based on a single trait model. Nevertheless, genetic evaluations are mostly carried out using a multi-trait model to take advantage of the genetic correlations among traits and estimate breeding values simultaneously. It can therefore be expected that traits with group measurements, can benefit from genetically correlated traits with individual measurements via multi-trait model (*e.g.*, FI and average daily gain).

## Conclusions

In conclusion, the proposed random regression model is feasible to handle repeated group measurements with consideration of drop out animals. In this study, we observed reduced VC estimates when animals were dropped out by ranking of phenotypes. However, using group measurements with drop out animals by ranking of phenotypes can provide similar accuracy but larger bias of EBV compared to those of using group measurements without dropped out animals. Therefore, it would be preferable to perform genetic evaluation using group measurements with VCs estimated from individual measurements. In addition, the findings clearly indicate that group composition plays a critical role in genetic evaluation when using group measurements. Hence, allocating animals with close genetic relationships to the same group could optimize the use of group measurements. Overall, this model could provide a cost-effective solution in breeding programs, thus it can be applied to the routine genetic evaluations and extended to more species and traits.
